# Identification of MADS-Box Transcription Factors in *Iris laevigata* and Functional Assessment of *IlSEP3* and *IlSVP* during Flowering

**DOI:** 10.3390/ijms23179950

**Published:** 2022-09-01

**Authors:** Guiling Liu, Fengyi Li, Gongfa Shi, Lei Wang, Ling Wang, Lijuan Fan

**Affiliations:** College of Landscape Architecture, Northeast Forestry University, Harbin 150040, China

**Keywords:** *Iris*, MADS-box, transcription factor, SEP3, SVP, flowering, *Arabidopsis*

## Abstract

*Iris laevigata* is ideal for gardening and landscaping in northeast China because of its beautiful flowers and strong cold resistance. However, the short length of flowering time (2 days for individual flowers) greatly limits its applications. Molecular breeding and engineering hold high potential for producing *I. laevigata* of desirable flowering properties. A prerequisite is to identify and characterize key flowering control genes, the identity of which remains largely unknown in *I. laevigata* due to the lack of genome information. To fill this knowledge gap, we used sequencing data of the *I. laevigata* transcriptome to identify MADS-box gene-encoding transcription factors that have been shown to play key roles in developmental processes, including flowering. Our data revealed 41 putative MADS-box genes, which consisted of 8 type I (5 Mα and 3 Mβ, respectively) and 33 type II members (2 MIKC* and 31 MIKC^C^, respectively). We then selected *IlSEP3* and *IlSVP* for functional studies and found that both are localized to the nucleus and that they interact physically in vitro. Ectopic expression of *IlSEP3* in *Arabidopsis* resulted in early flowering (32 days) compared to that of control plants (36 days), which could be mediated by modulating the expression of *FT*, *SOC1*, *AP1*, *SVP*, *SPL3*, *VRN1*, and *GA20OX*. By contrast, plants overexpressing *IlSVP* were phenotypically similar to that of wild type. Our functional validation of *IlSEP3* was consistent with the notion that *SEP3* promotes flowering in multiple plant species and indicated that *IlSEP3* regulates flowering in *I. laevigata.* Taken together, this work provided a systematic identification of MADS-box genes in *I. laevigata* and demonstrated that the flowering time of *I. laevigata* can be genetically controlled by altering the expression of key MADS-box genes.

## 1. Introduction

*Iris laevigata* (family of *Iridaceae*) is an herbaceous flowering plant species growing in the temperate regions of the Northern Hemisphere [1]. The large, bright, and elegant flowers, along with tall, verdant, and straight leaves, make *I. laevigata* of high ornamental values. In addition, *I. laevigata* is strongly tolerant of cold [2]. Thus, *I. laevigata* is an ideal gardening plant in the northeast region of China with high commercial value. Nonetheless, the potential of *I. laevigata* in gardening and landscaping is limited due to a short length of flowering time, typically only 2 days for an individual flower and 20–25 days for the population. Because the flower is the most important ornamental part of *I. laevigata*, it is critical for breeders to identify key factors controlling flowering time and use genetic tools to improve flowering traits [3].

Flowering is a critical developmental process featuring the transition from vegetative to reproductive growth in plants [4]. Molecular genetics have identified six major flowering pathways: the autonomous pathway, photoperiod pathway, gibberellic acid-dependent pathway, vernalization pathway, aging pathway, and ambient temperature pathway [4,5,6,7,8,9]. Extensive studies have demonstrated that these pathways form intricate networks that respond to a plethora of internal and external stimuli for flowering. Ultimately, these networks converge at several key floral pathway integrators such as *FLOWERING LOCUS T (FT)*, *SUPPRESSOR OF OVEREXPRESSION OF CONSTANS 1 (SOC1)*, *APETALA1 (AP1)*, and *FLOWERING LOCUS C (FLC)*. Much of detailed molecular works has been performed in the model plant *Arabidopsis* [10]. In *I. laevigata*, very few studies have been reported on the flowering control. Most studies used plants in the same genus, focusing on the characterization of the reproductive growth and modulation of flowering time via changing agronomic parameters, such as photoperiod and temperature, or using plant growth regulators [11,12,13,14]. Currently, the molecular identities of flowering pathway members and integrators in *I. laevigata* remain largely unknown.

In the model plant *Arabidopsis*, more than 100 genes involved in the flowering pathways have been characterized [10]. Among them, *agamous* has long been recognized as a key player in stamen and carpel development [15]. Sequence alignment showed that the *Arabidopsis agamous* gene encodes a transcription factor that is highly similar to homeotic proteins in yeast (Mini Chromosome Maintenance 1, or MCM1), snapdragon (Deficiens, or DEF), and humans (Serum Response Factor, or SRF). Collectively, these genes were named the MADS-box gene family using the first letter of the four founding gene members [16]. MADS-box transcriptional factors function in a diverse array of biological processes during development in eukaryotes. Nonetheless, all MADS-box proteins share the conserved MADS-box domain of around 60 amino acids in the N-terminus (also known as the M domain). Among them, MIKC-type genes encode plant-specific MADS-box proteins due to the presence of three additional domains (the intervening domain, keratin domain, and C-terminal domain) [17]. Two subtypes of MIKC, MIKC^C^, and MIKC*, have been recognized based on variances in the intervening domain [18].

Given the essential role of MADS-box transcription factors in flowering, extensive work has been performed in the identification of the encoding genes and delineation of gene functions [19,20,21,22,23,24,25,26]. Advances in genome sequencing also allowed whole-genome identification of MADS-box genes in many plant species. In the first comprehensive profiling of MADS-box genes, the consensus sequence encoding the MADS-box domain was used to search the genome of *Arabidopsis* [27]. MADS-box genes were identified, which were categorized into five groups (MIKC, Mα, Mβ, Mγ, and Mδ) based on phylogenetic analysis. Later, a similar approach was used to identify MADS-box genes in rice, another model plant for both basic and crop research [20]. Compared to *Arabidopsis*, the rice genome consists of a smaller MADS-box gene family with 75 members that are also categorized into five groups (MIKC^C^, MIKC*, Mα, Mβ, and Mγ). Since then, MADS-box genes have been documented in various plant species including bread wheat [21], willow [22], bamboo [23], watermelon [24], cucumber [25], and foxtail millet [26].

Due to the lack of genome information, molecular studies in *I. laevigata* are lagging far behind compared to those of other plant species. Consequently, little is known on the molecular mechanisms behind the flowering control in *I. laevigata*. In addition, the identity and function of MADS-box genes in *I. laevigata* have not been reported. To overcome the unavailability of genome data, our group recently performed RNA-sequencing using the flower tissue of *I. laevigata* [2]. The valuable resource made it possible to screen 23 R2R3-MYBs genes that are involved in cold-resistance in *I. laevigata* [2]. Using a similar approach, the aim of this study was to systematically identify MADS-box genes in *I. laevigata* and characterize selected genes in the context of flowering control. Here, we report the identification of 41 putative MADS-box genes and phylogenetic/structural analysis of candidate genes. We selected two genes for further functional studies and found that both *IlSEP3* (*SEPALLATA3*) and *IlSVP* (*SHORT VEGETATIVE PHASE*) encode proteins that are localized into the nucleus. Interestingly, the two proteins showed physical interactions. Overexpressing *IlSEP3*, but not *IlSVP*, promoted flowering in *Arabidopsis* possibly by regulating the expression of genes in multiple pathways.

## 2. Results

### 2.1. Genome-Wide Identification of MADS-Box Genes in I. laevigata

**Figure 1:**
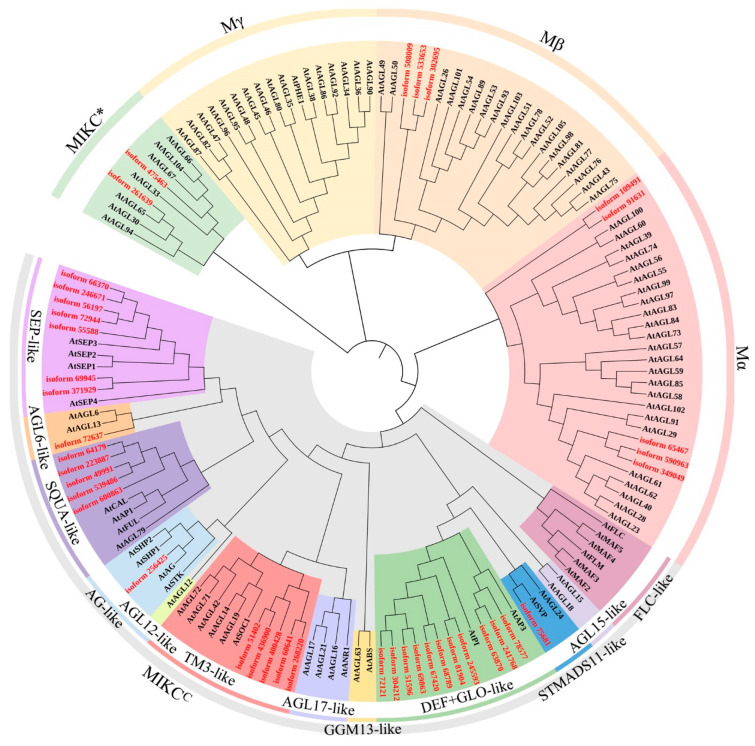
Phylogenetic analysis of MADS-box proteins. Red and black denote MADS-box proteins from *I. laevigata* and *Arabidopsis*, respectively.

A total of 41 non-redundant MADS-box genes were identified from our full-length transcriptomics database (Figure 1). Phylogenetic analysis showed that there are 8 type I and 33 type II MADS-box genes in *I. laevigata.* Among the 8 type I genes, 5 and 3 belong to the Mα and Mβ subfamily, respectively. Type II MADS-box genes consist of 2 MIKC* members and 31 MIKC^C^ members, which can be further divided into the following 7 subtypes: SEP-like (*n* = 7), AGL6-like (*n =* 1), SQUA-like (*n =* 5), AG-like (*n =* 1), TM3-like (*n =* 5), DEF/GLO-like (*n =* 11), and STMADS11-like (*n =* 1).

### 2.2. Cloning and Bioinformatics Analysis of IlSEP3 and IlSVP

**Figure 2:**
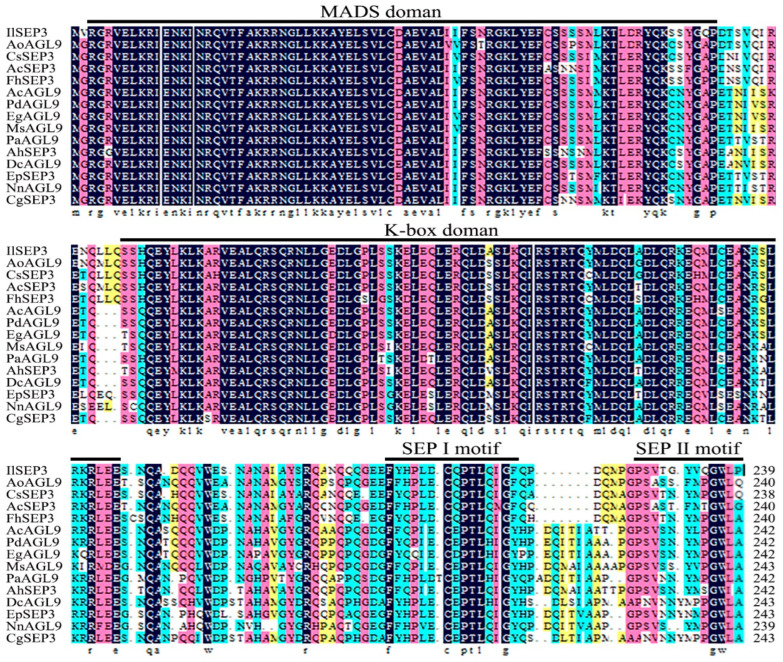
Sequence alignment of IlSEP3 proteins from different species. IlSEP3: *Iris laevigata* (ON398430); AoAGL9: *Asparagus officinalis* (XP_020247630.1); CsSEP3: *Crocus sativus* (ACB69509.1); AcSEP3: *Allium cepa* (QCT25556.1); FhSEP3: *Freesia* hybrid cultivar (QGV23785.1); AcAGL9: *Ananas comosus* (XP_020107646.1); PdAGL9: *Phoenix dactylifera* (XP_038986890.1); EgAGL9: *Elaeis guineensis* (XP_010913017.1); MsAGL9: *Musa acuminata* subsp. *Malaccensis* (XP_009415892.1); PaAGL9: *Persea americana* (AAX15924.1); AhSEP3: *Alpinia hainanensis* (ALB09087.1); DcAGL9: *Dendrobium catenatum* (XP_020706096.1); EpSEP3: *Euptelea pleiosperma* (ADC79706.1); NnAGL9: *Nelumbo nucifera* (XP_010250667.1); CgSEP3: *Cymbidium goeringii* (AHJ80843.1).

**Figure 3:**
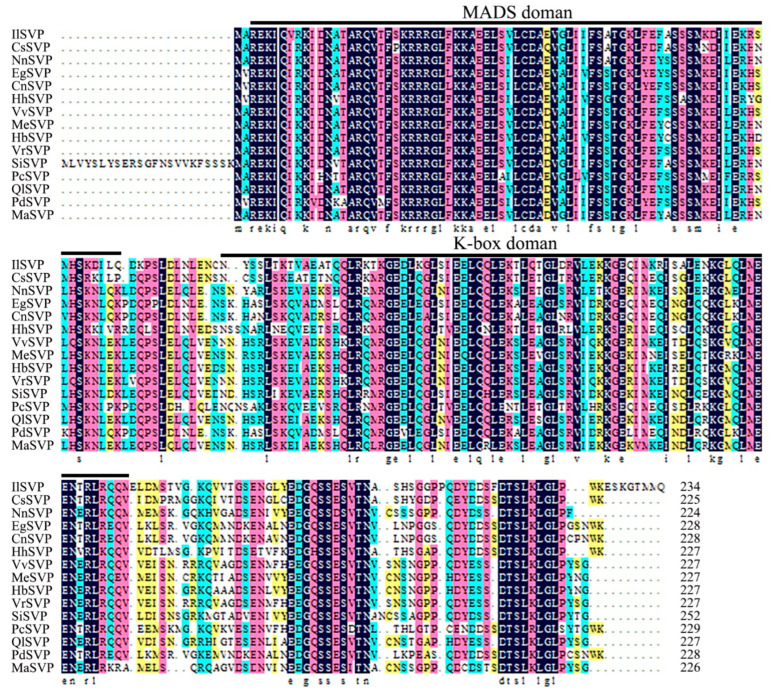
Sequence alignment of *Il*SVP proteins from different species. *Il*SVP: *Iris laevigata* (ON398431); *Cs*SVP: *Crocus sativus* (QIH12017.1); NnSVP: *Nelumbo nucifera* (XP_010254525.1); EgSVP: *Elaeis guineensis* (XP_010942683.1); CnSVP: *Cocos nucifera* (EHA8591139.1); HhSVP: *Hemerocallis* hybrid cultivar (QBX87860.1); VvSVP: *Vitis vinifera* (XP_019073897.1); MeSVP: *Manihot esculenta* (XP_021631111.1); HbSVP: *Hevea brasiliensis* (XP_021662492.1); VrSVP: *Vitis riparia* (XP_034692566.1); SiSVP: *Sesamum indicum* (XP_020554864.1); PcSVP: *Paphiopedilum callosum* (QXO37013.1); QlSVP: *Quercus lobata* (XP_030923627.1); PdSVP: *Phoenix dactylifera* (XP_038983281.1); MaSVP: *Morus alba* var. *alba* (AYK27567.1).

We next cloned both *IlSEP3* and *IlSVP* (Figure S1). Sequencing results confirmed that the ORF of *IlSEP3* was 720 bp, encoding a protein of 239 amino acid residues (Figure S2A). The ORF of *IlSVP* was 705 bp with a deduced protein of 234 amino acid residues (Figure S2B). Both genes were predicted to encode MIKC-type MADS-box proteins with the presence of a MEF2-like MADS domain and a K-box domain (Figure S3). Sequence alignment showed a high level of similarity between *Il*SEP3 and SEP3 from other plant species, all of which share the characteristic SEP3Ⅰmotif and SEP3Ⅱmotif (Figure 2). Similarly, a high level of sequence conservation was also found for *Il*SVP and its homologs from other plants (Figure 3). Phylogenetic analysis showed that the two proteins are most closely related to the counterparts in *Crocus sativus* (also in the *Iris* family, Figure S4).

The predicted physicochemical properties of both proteins were summarized in Table S1. Of note, both were predicted as unstable proteins (instability index > 40), indicative of a fast protein turnover. In addition, both showed negative values of the grand average of hydropathicity (GRAVY), suggesting that they are hydrophilic. Assessment on the hydrophobicity/hydrophilicity scale showed similar results with a significant portion of hydrophilic amino acid residues in both proteins (Figure S5). Prediction of the secondary structure showed that α-helix is the dominant form of both proteins (51.88% for *Il*SPE3 and 56.84% for *IL*SVP). Accordingly, the predicted tertiary structures also showed the dominance of α-helix (Figure S6). We also found multiple phosphorylation sites for both *Il*SEP3 (13 Ser,4 Thr, and 3 Tyr residues, Figure S7A) and *Il*SVP (17 Ser, 13 Thr, and 3 Tyr residues, Figure S7B). Finally, we did not identify any membrane-spanning domains or signaling peptides on either protein.

### 2.3. IlSEP3 and IlSVP Are Localized to the Nucleus

**Figure 4:**
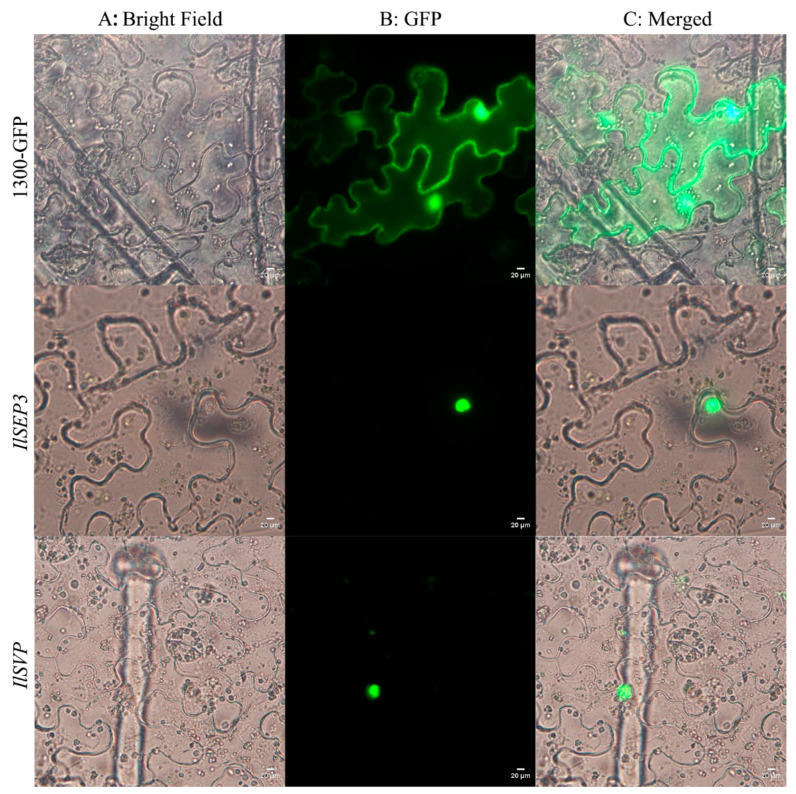
Subcellular localization of *Il*SEP3 and *Il*SVP. (**A**) Bright-field; (**B**) GFP; (**C**) merged signals.

Both *Il*SEP3 and *Il*SVP were predicted to function within the nucleus. This was validated by expressing GFP-fusion proteins transiently in *Nicotiana benthamiana* (Figure 4). Our data showed that both proteins were exclusively localized to the nucleus. By contrast, the empty vector harboring GFP showed non-specific autofluorescence in epidermal cells, especially in the cell membrane.

### 2.4. IlSEP3 Interacts with IlSVP

Information on the interacting proteins enables a better understanding of how protein act within a network. Unfortunately, the identity of interacting patterns for *Il*SEp3/*Il*SVP has rarely been reported. Thus, we used their homologs in the model plant *Arabidopsis* for this exploration. We found 20 interacting proteins for *At*SPE3 (Figure S8A). Many of them including SVP (others are AP1, LFY, FT, TFL1, AGL20, or SOC1) are involved in flowering control. For *At*SVP, we also found 20 interacting proteins including key regulators in the photoperiod pathway (CO and GI) and the circadian clock control including FKF1, ELF3, PRR7, TOC1, ZTL, DOF5.5, and COP1 (Figure S8B). Because of high sequence similarity, these data supported a role for *Il*SEP3 and *Il*SVP in flowering. In addition, interaction between IlSEP3 and IlSVP could be one mechanism by which these proteins control flowering.

**Figure 5:**
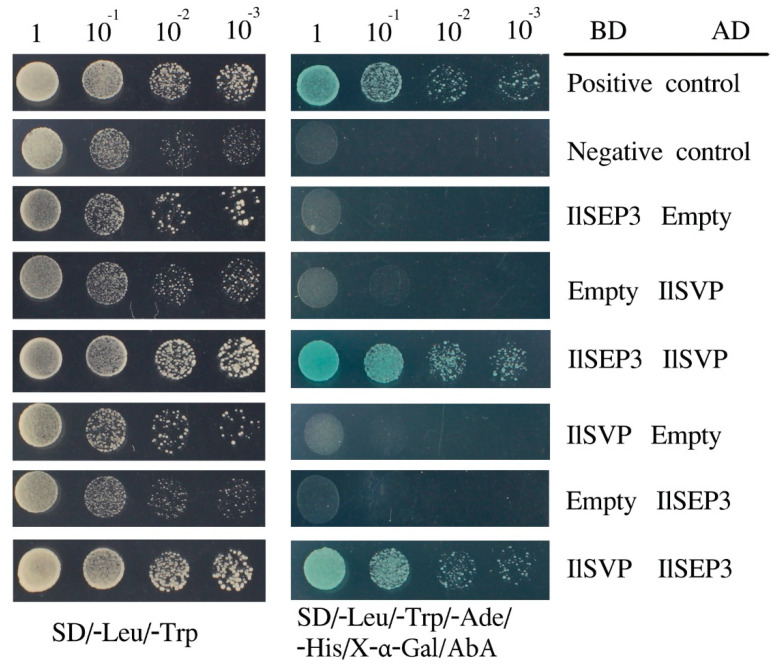
Interaction between IlSEP3 and IlSVP by yeast-two-hybrid. Numbers on top indicate dilution factors. The relative interaction strengths were determined by increasing dilution of yeast colonies. AD, active domain; BD binding domain. For co-transformation, eight groups were assessed: the test group (pGBKT7-IlSEP3 and pGADT7-IlSVP), (pGBKT7-IlSVP and pGADT7-IlSEP3), control for self-activation (pGBKT7-IlSEP3 and empty), (pGBKT7-IlSVP and empty), (empty and pGADT7-IlSVP), (empty and pGADT7-IlSEP3), positive control (pGADT7-T and pGBKT7-53), and negative control (pGADT7-T and pGBKT7-Lam). Transformed cells were diluted and then grown on either SD/-Trp/-Leu or SD/-Trp/-Leu/-Ade/-His/X-α-Gal/ABA at 28 °C for 48 h.

To validate the prediction, we constructed various of vectors for Y2H test. First, we transformed either pGBKT7-*IlSEP3* or pGBKT7-*IlSVP* alone into yeast cells and found no toxicity and autoactivation activities (Figure 5), Likewise, neither gene in the pGBKT7 vector can survive in the selective medium. We then co-transformed the two vectors into Y2HGold competent cells (Figure 5). We found that strains harboring IlSEP3-IlSVP can grow on the selective medium (SD/-Trp/-Leu/-Ade/-His). As expected, the positive control harboring pGADT7-T and pGBKT7-53 also showed colony formation on the high stringent medium. By contrast, no colony was seen for the negative control harboring pGADT7-T and pGBKT7-Lam. These data indicated that *Il*SEP3 interacts with *Il*SVP physically.

### 2.5. Ectopic Expression of IlSEP3 in Arabidopsis Promotes Early Flowering

**Figure 6:**
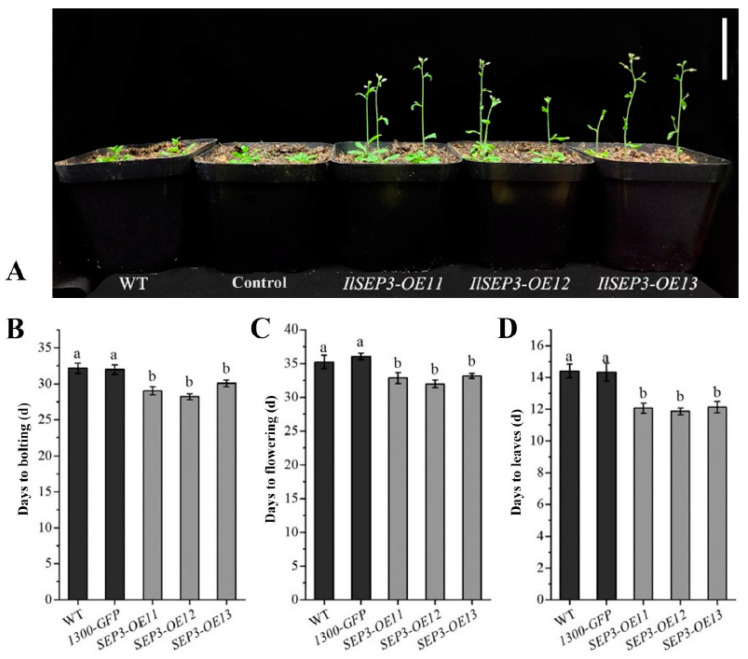
Phenotypes of transgenic *Arabidopsis* overexpressing *IlSEP3*. (**A**) Representative plants grown under long-day conditions. Bar = 10 cm. (**B**) Days to bolting (inflorescence stalk of 1 cm). (**C**) Days to flowering (opening of the first flower). (**D**) The number of rosette leaves at the time of flowering. All data represent mean ± standard error. Statistical difference was denoted by lowercase letters (*p* < 0.05).

To test the functional relevance of the two selected MADS-box genes, we created transgenic *Arabidopsis* overexpressing *IlSEP3* or *IlSVP*. We obtained 15 transgenic lines for *IlSEP3* and 16 lines for *IlSVP* after antibiotic selection (Hygromycin) and PCR screening (Figure S9). Three overexpressing lines (OE) for each were selected for phenotypical study. As expected, plants transformed with the empty vector were phenotypically identical to that of wild type (Figure 6). Compared to these controls, plants overexpressing *IlSEP3* showed significant early bolting and flowering (Figure 6A–C). While the controls bolted and flowered at 32 d and 36.08 d, respectively, the *IlSEP3*-OE12 line showed a bolting time of 28.2 d and a flowering time of 32 d (Figure 6B,C). In addition, the number of rosette leaves is another important indicator for plant development and transition to reproductive growth. Our data showed fewer leaves in the transgenic plants (mean of 11.93) compared to the controls (mean of 14.38) at the time of flowering (Figure 6D).

Phenotypical analysis of three *IlSVP* lines showed a slightly early flowering time (34.5, 34.3, and 34.3 d, respectively) compared to the controls (mean of 36.08 d). However, the difference was not statistically significant (*p* > 0.05). Thus, plants overexpressing *IlSVP* were not used for further experiments in this study.

### 2.6. Overexpression of IlSEP3 Modulates the Expression of Flowering Time Genes

**Figure 7:**
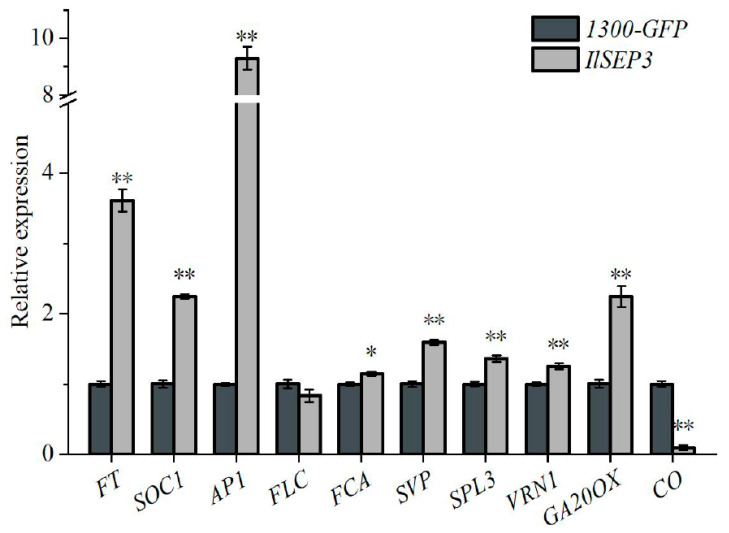
Expression of endogenous flowering time genes in transgenic *Arabidopsis* overexpressing *IlSEP3*. Data represent mean ± standard error. Statistical difference was denoted by * (*p* < 0.05) or ** (*p* < 0.01).

To understand the molecular mechanism by which *IlSEP3* promotes early flowering, the mRNA abundance of various endogenous flowering time genes was determined in the transgenic plants (Figure 7). Compared to plants transformed with the empty vector, overexpression of *IlSEP3* resulted in significant up-regulation of *FT*, *SOC1*, *AP1*, *SVP*, *SPL3*, *VRN1*, and *GA20OX*. By contrast, CO was significantly down-regulated in the transgenic plants. In addition, the expression of *FLC* was comparable between the transgenic plants and the control.

## 3. Discussion

In this study, we identified 41 MADS-box genes in *I. laevigata* using transcriptomics data. The number of genes is significantly less than that in *Arabidopsis* (*n* = 107). However, our data is comparable to 39 MADS-box genes in the Louisiana Irises (*Iris fulva*), 25 of which belonged to the MIKC^C^ type [28]. Notably, transcriptomics data generated from floral and young leaf tissues were also used for the Louisiana Irises study due to the lack of genome sequencing data. Similarly, 43 MADS-box genes were identified in the de novo transcriptomics study of *Iris atropurpurea*, an endemic species in Israel [29]. Compared to 33 MIKC^C^ genes identified in this study, 28 were found in the *I. atropurpurea* transcriptome. Thus, these data suggested similar gene numbers and subtypes among closely related *Iris* species.

Currently, the MIKC^C^ subgroup members are the most extensively studied MADS-box genes. Based on phylogenetic analysis, 12 subgroups have been found in *Arabidopsis* [30]. Nonetheless, many subgroups, including *FLC*-like, *AGL15*-like, *GGM13*-like, *AGL17*-like, and *AGL12*-like, were not identified in *I. laevigata*. This is consistent with a previous report, where *FLC*-like, *GGM13*-like, *TM8*-like, *AGL15*-like, *AGL17*-like, and *AGL12*-like were not identified in the Louisiana Irises [28]. Lacking some of these subgroups in *Iris* is supportive of the notion that *FLC*-like is specific to dicots such as *Arabidopsis* [20,28,30]. By contrast, the lack of other subgroups in our transcriptome data could be attributed to spatial gene expression. For instance, *AGL15*-like, *GGM13*-like, *AGL17*-like, and *AGL12*-like showed high mRNA expression levels in the root [30]. The use of floral and leaf tissues for transcriptome data generated in this study cannot rule out the possibility that these MADS-box subgroups may exist in the genome. It would be interesting to compare these data to putative MADS-box genes from genome sequencing data once available. Such comparisons can reveal a more comprehensive picture of MADS-box genes in *I. laevigata*.

For functional studies, we selected two members in the MIKC^C^ branch. Both *IlSEP3* (belonging to the *SEP*-like sub-branch) and *IlSVP* (belonging to the STMADS11-like sub-branch) showed high sequence similarities to their corresponding homologs in other plant species. The physical interactions between *IlSEP3* and *IlSVP* indicated that they may function as a complex in vivo regulating flowering. This challenges the conventional view that *SVP* and *SEP3* function in the early stage of flowering initiation and floral organ formation, respectively [31]. Instead, it supports the emerging notion that these genes form an integrated network to control the flowering process [32]. Experimental evidence in favor of the later model have been provided by yeast three-hybrid showing the formation of higher-order complexes among MADS-box proteins with SEP3 serving as a glue for multimerization [31,32,33,34]. Further in vivo studies are needed to validate the interaction of SEP3 with other proteins for flowering control.

Our overexpression experiments provided a direct assessment of molecular functions of *IlSEP3* and *IlSVP*, which showed that *IlSEP3* promotes flowering. This is consistent with previous studies in which an earlier flower phenotype was observed in transgenic *Arabidopsis* overexpressing *SEP3* from different plant species including lavender [35], *Ziziphus jujuba* Mill. [36], lily [37], and woad (*Isatis indigotica*) [38]. Conversely, silencing pf *IiSEP3-2* and *IiSEP3-3* using virus-induced gene silencing (VIGS) resulted in delayed flowering in woad [38]. In addition to flowering, overexpressing *SPE3s* in *Arabidopsis* often resulted in curly and smaller leaves compared to WT. Thus, our results and data from others strongly support that SPE3 is a positive player in flowering.

Mechanistically, we found that overexpressing *IlSEP3* led to perturbed expression patterns of key flowering genes including *FT*, *SOC1*, *AP1*, *VRN1*, *AG20OX*, as well *CO* (downregulation). Previous studies also showed that overexpressing of these upregulated genes (*FT*, *SOC1*, *AP1*, *VRN1*, and *AG20OX*) promotes flowering [39,40,41]. These data also indicated that *IlSEP3* can modulate various flowering pathways, including the gibberellic acid-dependent pathway and the vernalization pathway in promoting early flowering in transgenic plants. It is also noteworthy that overexpressing *IlSEP3* resulted in a higher expression of SVP. A similar repressuring effect on SVP has been found in *Arabidopsis* plants overexpressing either the *SEP3-2* or *SEP3-3* splicing variant of woad [38]. More interestingly, *SVP* was upregulated before flowering and downregulated after flowering in *Arabidopsis* plants overexpressing *ZjSEP3* [36]. It is possible that the observed gene expression pattern is dependent on the developmental stage given dynamic interactions among these genes/pathways. A time-course gene expression study would be required to clarify the exact regulatory network in the *IlSEP3-*overexpression plants.

By contrast, we found no impact on flowering time by overexpressing *IlSVP*. Although *SVP* is often considered a repressor of flowering, the literature is divided on its molecular function. For example, ectopic expression of kiwifruit *SVP3* (*Actinidia spp.*) in *A. deliciosa* showed no impact on flower time but led to prolonged flowers [42]. TaVrt2, an SVP-like MADS-box protein, has been demonstrated to promote flowering in the vernalization pathway via binding to the promoter of *TaVrn1* (encoding another MADS-box transcription factor) [43]. In addition, overexpression of *SVP* from sweet cherry resulted in a delay in flowering and the production of flowers with curly sepals in *Arabidopsis* [44]. Thus, it seems the exact function of SVP is plant species dependent. Currently, the mechanism underlying the functional divergence of *SVPs* among different plant species remains unclear. Nonetheless, *SVP* may represent a gene characterized with functional variations as SVPs from the same species can exhibit distinct functions. For instance, the four SVPs in kiwifruit showed gene-specific expression patterns and functions during vegetative growth, flowering, and bud dormancy [42]. The duality of conservation and divergence of *SPVs* have also been observed in *Arabidopsis*, in which *SVP* and *SGL24* can function in concert with each other during one developmental stage (e.g., flower development) but perform opposite roles for another process (e.g., floral initiation) [45,46]. In terms of functional conservation among different species, further studies can be performed by expressing *IlSVP* in the corresponding *Arabidopsis* mutant to see whether it can rescue the *svp* mutant.

## 4. Materials and Methods

### 4.1. Gene Sequence Identification and Phylogenetic Analysis

An annotated transcriptomics dataset of *I. laevigata* (unpublished from our group) was used to identify the MADS-box gene family. The open reading frame (ORF) of the obtained sequences was predicted by the TBtools (https://github.com/CJ-Chen/TBtools, accessed on 1 January 2022). Domain analysis was performed using the online Batch CD-Search tool (https://web.expasy.org/protparam/, accessed on 1 January 2022). Sequences harboring the MADS-box domain were kept and subjected to a second-round ORF analysis (https://www.ncbi.nlm.nih.gov/orffinder/, accessed on 1 January 2022) to ensure the integrity of the transcript.

Phylogenetic analysis was performed using MEGA X with MADS-box sequences from *I. laevigata* and *Arabidopsis*. MADS-box proteins of *Arabidopsis* were downloaded from Phytozome (https://phytozome-next.jgi.doe.gov/, accessed on 1 January 2022). Sequence alignment was performed using Clustal W, and the neighbor-joining (NJ) method was used for tree construction with a bootstrap of 1000 times. Annotation of the phylogenetic tree was performed using iTOL (https://itol.embl.de/, accessed on 1 January 2022).

### 4.2. Gene Cloning and Bioinformatics

Total RNA was extracted from the leaf and flower of *I. laevigata* plants grown in the nursery of Northeast Forestry University (May 2020) using a kit (KANGWEI). Agarose gel electrophoresis was used to check the quality of the RNA, and DNA Eraser was used to remove genomic DNA. Next, 1 µg of total RNA was used for reverse transcription (TaKaRa). Amplification of target genes was performed using PCR with the following primers: 5′-TATTCAATGGTGAGGGGGAGAGTGGAGC-3′ and 5′-TTATGTGCAGGGATGGCTTCCGTGAGAC-3′ for *IlSEP3*; and 5′-TCTTTCTCCTCTGTTGCTGTGT-3′ and 5′-GAAGCTCTACTGCATCATCGTG-3′ for *IlSVP*. The obtained gene fragments were ligated into the pEASY-Blunt Zero Cloning Vector (TRANSS) and transformed into *E. coli*. Positive clones were selected for sequencing (QINGKE). All procedures were performed per instructions of respective kits.

Physical and chemical parameters were predicted using the ProtParam tool (https://web.expasy.org/protparam/, accessed on 1 January 2022). The hydrophobicity/hydrophilicity scale was assessed using ProtScale (https://web.expasy.org/protscale/, accessed on 1 January 2022). Prediction of the secondary and tertiary structures was performed using SOPMA and SWISS-MODEL, respectively. Potential phosphorylation sites were predicted by the NetPhos software. Subcellular localization was predicted using Wolf Psort.

### 4.3. Yeast-Two-Hybrid (Y2H)

To construct vectors for Y2H, the coding sequences of *IlSEP3* and *IlSVP* were first amplified by PCR. Plasmids containing pEASY-*IlSEP3* or pEASY-*IlSVP* were used as template. Primers for *IlSEP3* into pGBKT7 were 5′-CATATGTATTCAATGGTGAGGGGGAGAGTGGAGC-3′ (*Nde*I for restriction site, underlined) and 5′-GGATCCGTCCGGAAGCCATCCCTGCACATAACCA-3′ (*BamH*I). Primers for *IlSEP3* into GADT7 were 5′-ATATGGCCATGGAGGCCAGTGAATTCATTCAATGGTGAGGGGGAGAGTGGAGC-3′ and 5′-ATCTGCAGCTCGAGCTCGATGGATCCGTCCGGAAGCCATCCCTGCACATAACCA-3′ (homology arm, underlined). Primers for *IlSVP* into pGBKT7 were 5′-ATATGGCCATGGAGGCCAGTGAATTCATGGCGAGGGAGAAGATACAG-3′ (homology arm, underlined) and 5′-ATCTGCAGCTCGAGCTCGATGGATCCCTGCATCATCGTGCCCTTC-3′. Primers for *IlSVP* into pGADT7 were 5′-CCATGGAGGCCAGTGAATTCATGGCGAGGGAGAAGATACAG-3′(homology arm, underlined) and 5′-AGCTCGAGCTCGATGGATCCCTGCATCATCGTGCCCTTC-3′. pGBKT7-*IlSEP3* was obtained by subcloning *IlSEP3* into the pre-linearized pGBKT7 vector (*Nde*I and *BamH*I). pGADT7-*IlSEP3* was obtained by subcloning *IlSEP3* into GADT7 via (*EcoR*I and *BamH*I) homologous recombination using the ClonExpress II One Step Cloning Kit. In the same way, pGADT7-*IlSVP* (*EcoR*I and *BamH*I) and pGBKT7-*IlSVP*(*EcoR*I and *BamH*I) were obtained.

The Y2HGold yeast was used as the bait strain. Transformation into the competent cells was performed as described before [47]. Toxicity test was performed by transforming pGBKT7-*IlSEP* or pGBKT7-*IlSVP* into Y2HGold and growing the cells on SD/-Trp. For the autoactivation test, cells transformed with pGBKT7-*IlSEP* or pGBKT7-*IlSVP* were grown on SD/-Trp/X-α-gal/AbA. For co-transformation, eight groups were assessed: the test group (pGBKT7-*IlSEP3* and pGADT7-*IlSVP*), (pGBKT7-*IlSVP* and pGADT7-*IlSEP3*), (pGBKT7-*IlSEP3* and empty), (pGBKT7-*IlSVP* and empty), (empty and pGADT7-*IlSVP*), (empty and pGADT7-*IlSEP3*), positive control (pGADT7-T and pGBKT7-53), and negative control (pGADT7-T and pGBKT7-Lam). Transformed cells were diluted and then grown on either SD/-Trp/-Leu or SD/-Trp/-Leu/-Ade/-His/X-α-Gal/ABA at 28 °C for 48 h.

### 4.4. Vector Construction for Protein Expression

For gene overexpression, pCAMBIA1300-Pro35S: *IlSEP3-GFP* and pCAMBIA1300-Pro35S: *IlSVP--GFP* vectors were constructed by homologous recombination. Briefly, the full-length coding region was amplified using the following primers with homology arms (underlined): 5′-TTGATACATATGCCCGTCGACTATTCAATGGTGAGGGGGAGAGTGGAGC-3′ and 5′-CCCTTGCTCACCATGGATCCGTCCGGAAGCCATCCCTGCACATAACCA-3′ for *IlSEP3*; and 5′-TTGATACATATGCCCGTCGACTCTTTCTCCTCTGTTGCTGTGT-3′ and 5′-CCCTTGCTCACCATGGATCCGAAGCTCTGCATCATCGTGCCC-3′ for *IlSVP*. These constructs, along with the empty vector (pCAMBIA1300-Pro35S: *GFP*, used as a negative control), were then delivered into *Agrobacterium tumefaciens* GV3101 by the freeze-and-thaw method [48]. Positive clones were confirmed by sequencing.

### 4.5. Subcellular Localization

Subcellular localization was performed by transient expression of the *IlSEP3-GFP* and *IlSVP-GFP* proteins in *Nicotiana benthamiana* leaves according to previously established methods [49]. Briefly, positive clones harboring pCAMBIA1300-Pro35S: *IlSEP3-GFP* or pCAMBIA1300-Pro35S: *IlSVP--GFP* were cultured. Bacterial cells were collected by centrifuge and resuspended in 10 mM MaCl_2_ to OD_600_ of 1.5, followed by the addition of acetosyringone to a final concentration of 200 µM. After activation at dark for 3 h, the bacterial solution was injected into the downside of the leaf using a syringe. Fusion proteins in the epidermal cells of the transformed plants were determined under microscopy.

### 4.6. Overexpression of IlSEP3 and IlSVP in Arabidopsis

Transformation of *A. thaliana* (ecotype Col-0) was performed using the floral dipping method [50]. Seeds of the transformed plants were selected on 1/2 MS medium supplemented with 25 mg/L Hyg. After 7–9 days, T1 seedlings that can grow on the selective medium were transferred to the potting mixture and further cultured under 16 h/8 h (light/dark) at 20 °C. Transgenic plants were also screened by PCR. Homozygous lines were obtained by successive screening for three generations. The expression level of *IlSEP3* and *IlSVP* were determined by qRT-PCR using the seeding leaf of the transgenic plants. Actin2 (AT3G18780) was used as a control. Relative gene expression was determined using the 2^−ΔΔCT^ method. Three lines of a high level of expression were selected.

### 4.7. Phenotypical Analysis

Phenotypical analyses were performed as described previously [51,52,53]. Transgenic plants, wild-type Col-0, and plants transformed with the empty vector (Pro35S: GFP) were compared. Seeds were germinated on 1/2 MS plates and seedlings were transferred to potting soil after 2 weeks. Plants were allowed to grow at long-day conditions (16 h light/8 h dark) 20 °C. Bolting was defined when the inflorescence stalk is 1 cm long. Flowering was defined as the opening of the first flower. The number of rosette leaves was recorded at the flowering time. For each line, data from 12 individual plants were collected.

### 4.8. Expression of Flowering Genes

The expression of 10 well-known genes in flowering control (*CO*, *GA20OX*, *VRN1*, *SPL3*, *FCA*, *SVP*, *FT*, *SOC1*, *AP1*, and *FLC*) was determined by RT-PCR. Two-week-old seedlings were used. The selection of these genes and the time for gene expression analysis were based on previous studies [54,55,56,57,58]. Relative expression against the *AtACT2* control (2^−ΔΔCT^ method) was shown.

### 4.9. Statistical Analyses

Student’s *t* test was performed for phenotypical data and relative gene expression results. Statistical difference was determined between the transgenic and the control plants. Bar plots were used for data presentation.

## 5. Conclusions

In summary, we identified 41 putative MADS-box genes in the *I. laevigata* flower transcriptome. The putative MADS-box gene family may expand upon the availability of *I. laevigata* genome due to spatial-specific gene expression. Ectopic expression of *IlSEP3* in *Arabidopsis* promotes flowering by regulating various flowering pathways. The impact of overexpressing IlSEP*3* in *I. laevigata* on flowering awaits further investigation. If successful, the discovery here could broaden the application of *I. laevigata* in the wetlands of northeastern China by providing a genetic resource for flowering time control.

It would be intriguing to generate transgenic *I. laevigata* overexpressing *IlSEP3* and evaluate the impact on flowering. Based on the current results, one would reason an early flowering phenotype in the transgenic *I. laevigata* plants. If this indeed is the case, a broader application of *I. laevigata* for landscaping/gardening in northeastern China can be expected. It can be even imagined that this finding can be used to prolong the overall length of flowering by planting early-flowering transgenic plants along with wild-type plants. If a relationship between gene dosage and flowering time exists, it can be further exploited to create *I. laevigata* plants of the most ideal flowering time. Flowering in spring (e.g., May) would be beneficial because of a general lacking flowering plants in wetland during that time window of the year.

## Data Availability

Data from this study are available from the corresponding author upon reasonable request.

## Supplementary Materials

The supporting information can be downloaded at: https://www.mdpi.com/article/10.3390/ijms23179950/s1.
