# Supplementary material for: Identification of MADS-Box Transcription Factors in Iris laevigata and Functional Assessment of IlSEP3 and IlSVP during Flowering
Source: Int J Mol Sci. 2022 Sep 1;23(17):9950. doi: 10.3390/ijms23179950 (PMC9456522; doi:10.3390/ijms23179950)

Supplemental Figures

Supplemental Figure S1.

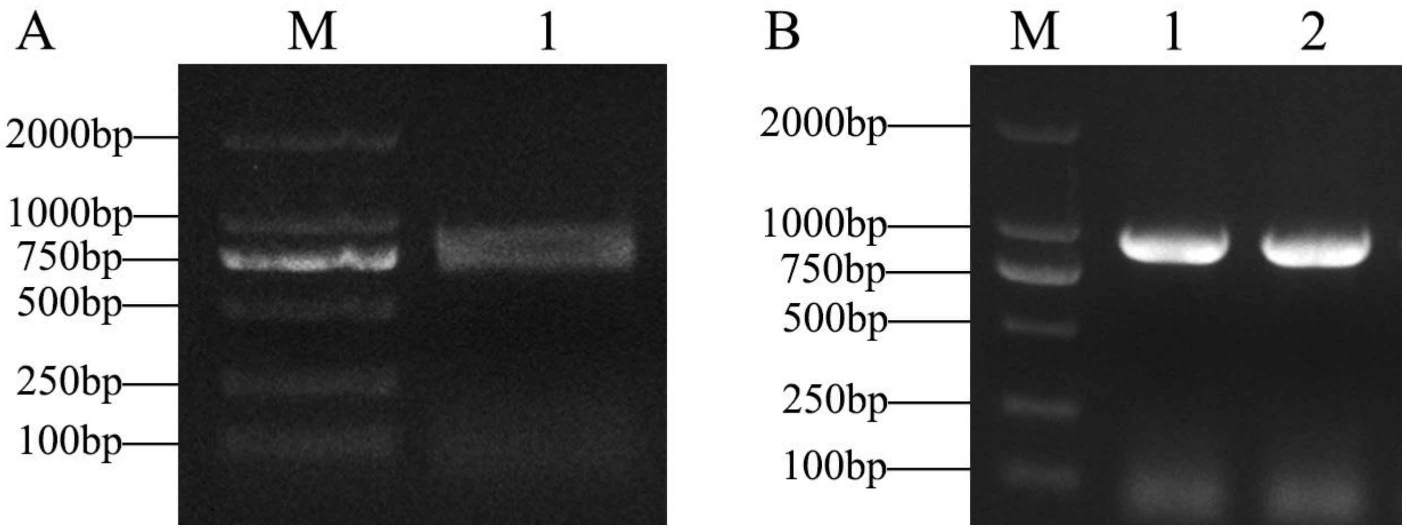

Supplemental Figure S2.

A

```
1    ATGGTGAGGGGGAGAGTGGAGCTGAAGAGGATTGAGAACAAGATCAACCGGCAGGTGACATTCGCCAAGAGGAGGAATGGGCTGCTCAAG
1     M  V  R  G  R  V  E  L  K  R  I  E  N  K  I  N  R  Q  V  T  F  A  K  R  R  N  G  L  L  K

91   AAGGCCTACGAGCTGTCTGTGCTCTGCGACGCCGAGGTCGCTCTCATCATCTTCTCCAACCGAGGGAAGCTCTATGAGTTCTGCAGCAGC
31    K  A  Y  E  L  S  V  L  C  D  A  E  V  A  L  I  I  F  S  N  R  G  K  L  Y  E  F  C  S  S

181  TCCAGCATGTTGAAAACACTGGATAGGTATCAAAAAAGCAGTTATGGGCAACCTGATACCAGTGTACAAATAAGGGAGAATCAGTTGCTG
61    S  S  M  L  K  T  L  D  R  Y  Q  K  S  S  Y  G  Q  P  D  T  S  V  Q  I  R  E  N  Q  L  L

271  CAGAGTAGTCATCAAGAATACTTGAAACTCAAAGCGCGTGTGGAAGCTTTACAACGATCACAAAGGAATCTTCTTGGTGAGGATCTTGGG
91    Q  S  S  H  Q  E  Y  L  K  L  K  A  R  V  E  A  L  Q  R  S  Q  R  N  L  L  G  E  D  L  G

361  CCTCTAAGCAGCAAGGAGCTGGAGCAACTCGAAAGACAACTAGACGCATCGTTAAAGCAAATAAGATCAACAAGGACACAGTATATGCTC
121   P  L  S  S  K  E  L  E  Q  L  E  R  Q  L  D  A  S  L  K  Q  I  R  S  T  R  T  Q  Y  M  L

451  GACCAGCTTGCTGACCTTCAAAGAAAGGAACAGATGCTCTGTGAAGCTAACAGAAGTCTCAGGAAGAGGTTGGAAGAAAGTAACCAGGCT
151   D  Q  L  A  D  L  Q  R  K  E  Q  M  L  C  E  A  N  R  S  L  R  K  R  L  E  E  S  N  Q  A

541  GACCAGCAGGTCTGGGAGTCCAATGCTAATGCAATAGCATACAGTCGGCAAGCAAATCAACAACAAGGAGAAGAATTCTACCACCCTCTA
181   D  Q  Q  V  W  E  S  N  A  N  A  I  A  Y  S  R  Q  A  N  Q  Q  Q  G  E  E  F  Y  H  P  L

631  GATTGCCAGCCCACTCTACAGATTGGGTTCCAACCTGATCAAATGCCTGGTCCGAGTGTGACTGGTTATGTGCAGGGATGGCTTCCGTGA
211   D  C  Q  P  T  L  Q  I  G  F  Q  P  D  Q  M  P  G  P  S  V  T  G  Y  V  Q  G  W  L  P  *
```

B

```
1    ATGGCGAGGGAGAAGATACAGGTGCGGAAGATTGACAACGCGACGGCGAGGCAGGTGACCTTCTCGAAGAGGAGGAGGGGGCTGTTCAAG
1     M  A  R  E  K  I  Q  V  R  K  I  D  N  A  T  A  R  Q  V  T  F  S  K  R  R  R  G  L  F  K

91   AAGGCCGAGGAGCTCTCCGTCCTCTGCGACGCCGAGGTCGGGCTCATCATCTTCTCCGCCACCGGCAAGCTCTTCGAGTTCGCCAGTTCC
31    K  A  E  E  L  S  V  L  C  D  A  E  V  G  L  I  I  F  S  A  T  G  K  L  F  E  F  A  S  S

181  AGTATGAAAGATATCATTGAAAAGCGCAGCATGCATTCAAAGGACATATTGCAGGACAAACCATCACTGGACTTGAATTTAGAGAATTGT
61    S  M  K  D  I  I  E  K  R  S  M  H  S  K  D  I  L  Q  D  K  P  S  L  D  L  N  L  E  N  C

271  AATTATTCAAGTCTGACAAAAACAGTCGCGGAGGCAACTCAACAACTAAGAAAGACGAAGGGTGAAGACCTTAAAGGCTTATCGATAGAG
91    N  Y  S  S  L  T  K  T  V  A  E  A  T  Q  Q  L  R  K  T  K  G  E  D  L  K  G  L  S  I  E

361  GAGCTTCAACAGCTAGAGAAAACTCTTCAAACTGGGTTAGATCGAGTTCTAGAGAAAAAGGGTGAGCAGATTATGAAAAGGATCAGTGCT
121   E  L  Q  Q  L  E  K  T  L  Q  T  G  L  D  R  V  L  E  K  K  G  E  Q  I  M  K  R  I  S  A

451  CTTGAAAATAAGGGACTCCAATTAATGGAAGAGAATACACGACTGAGACAACAAATGGAGCTCGATATGTCGACAGTGGGAAGCAGGTT
151   L  E  N  K  G  L  Q  L  M  E  E  N  T  R  L  R  Q  Q  M  E  L  D  M  S  T  V  G  K  Q  V

541  GTTACCGGCTCTGAAAATGGATTATATGAAGATGGACAGTCCTCAGAATCAGTCACCAACGCTTCACATTCTGGGGGCCGCCTCAAGAT
181   V  T  G  S  E  N  G  L  Y  E  D  G  Q  S  S  E  S  V  T  N  A  S  H  S  G  G  P  P  Q  D

631  TATGATGACAGTTTTGACACCTCTCTCAAGTTAGGGCTTCCGTGGAAGGAATCGAAGGGCACGATGATGCAGTAG
211   Y  D  D  S  F  D  T  S  L  K  L  G  L  P  W  K  E  S  K  G  T  M  M  Q  *
```

Supplemental Figure S3.

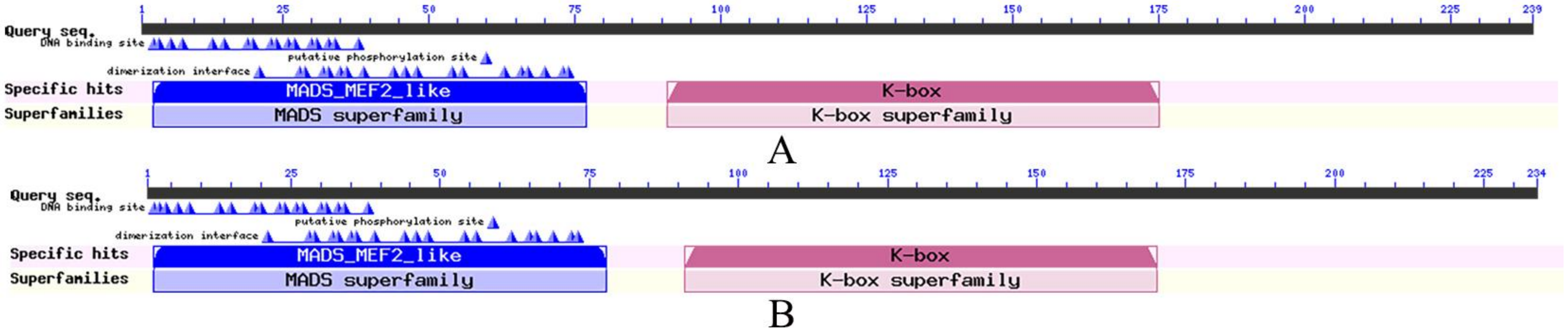

Supplemental Figure S4.

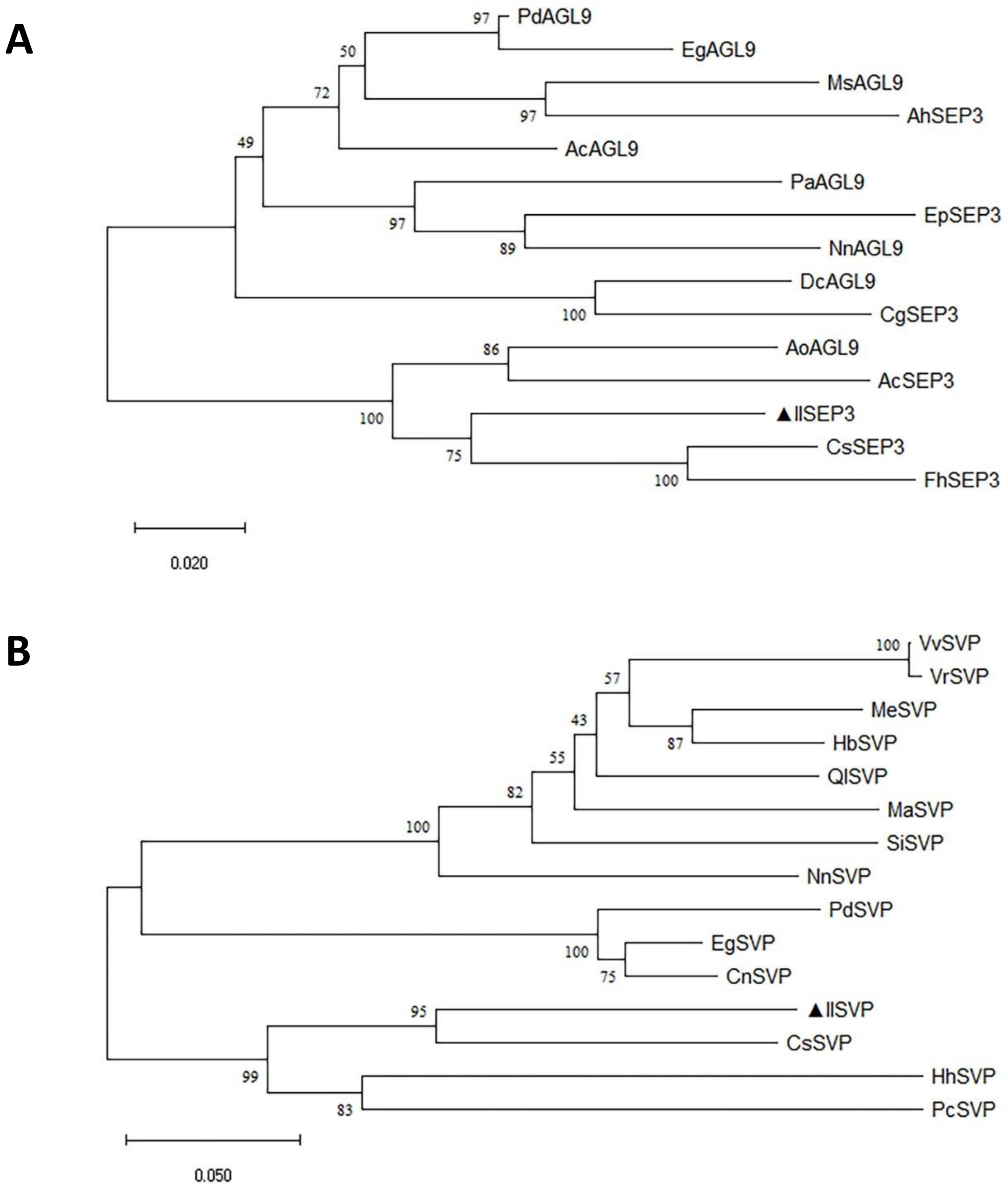

Supplemental Figure S5

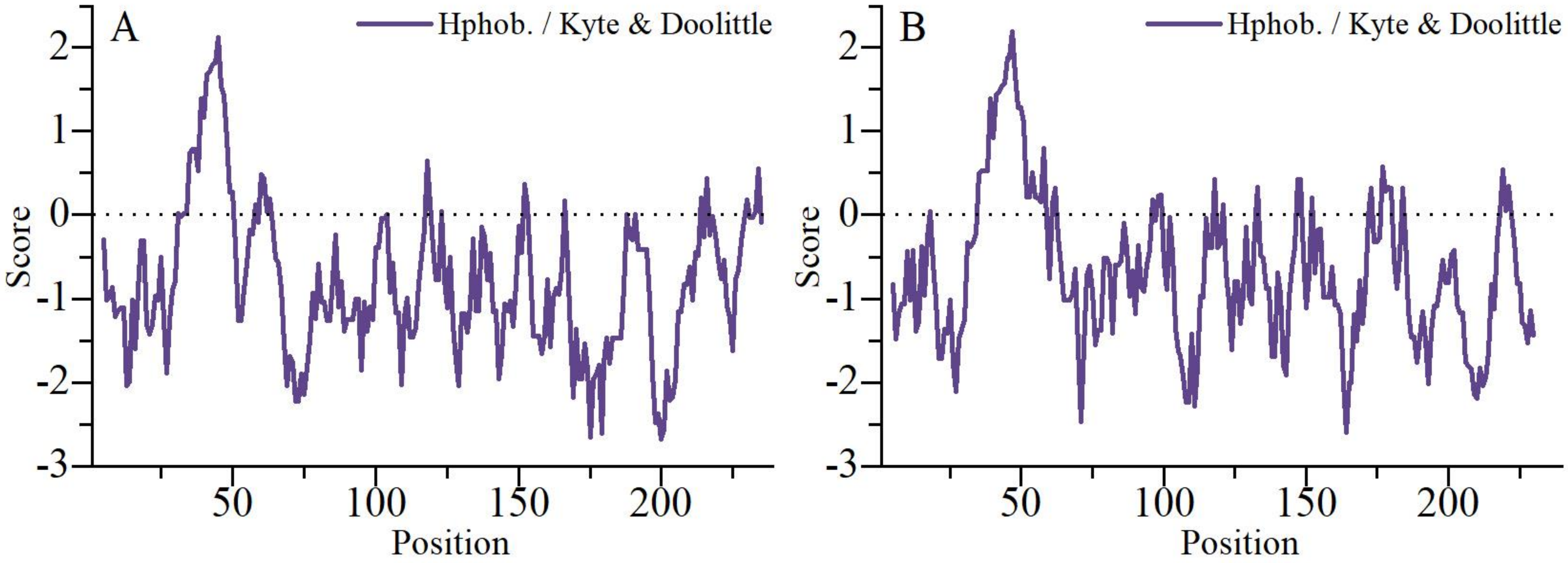

Supplemental Figure S6

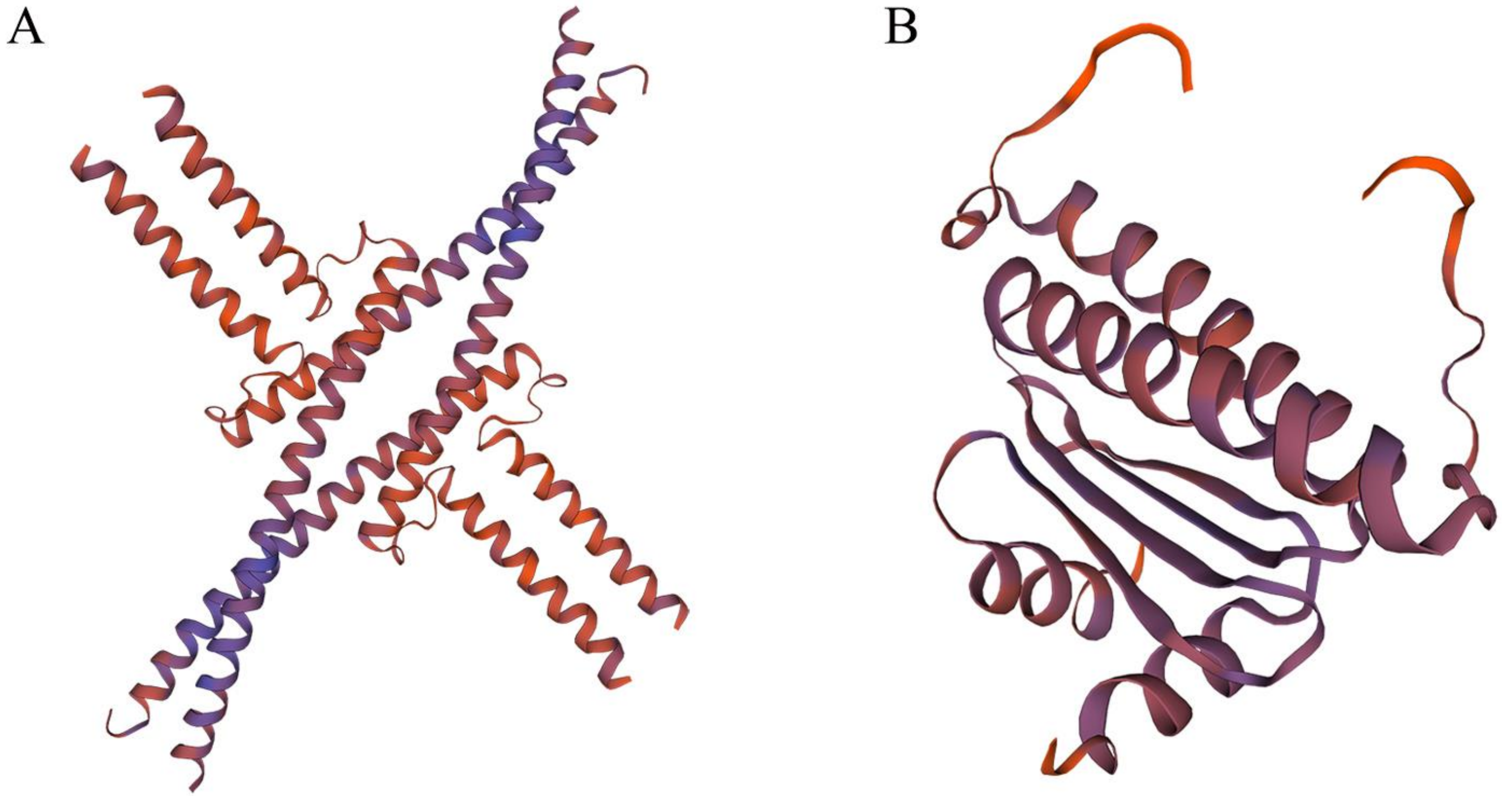

Supplemental Figure S7

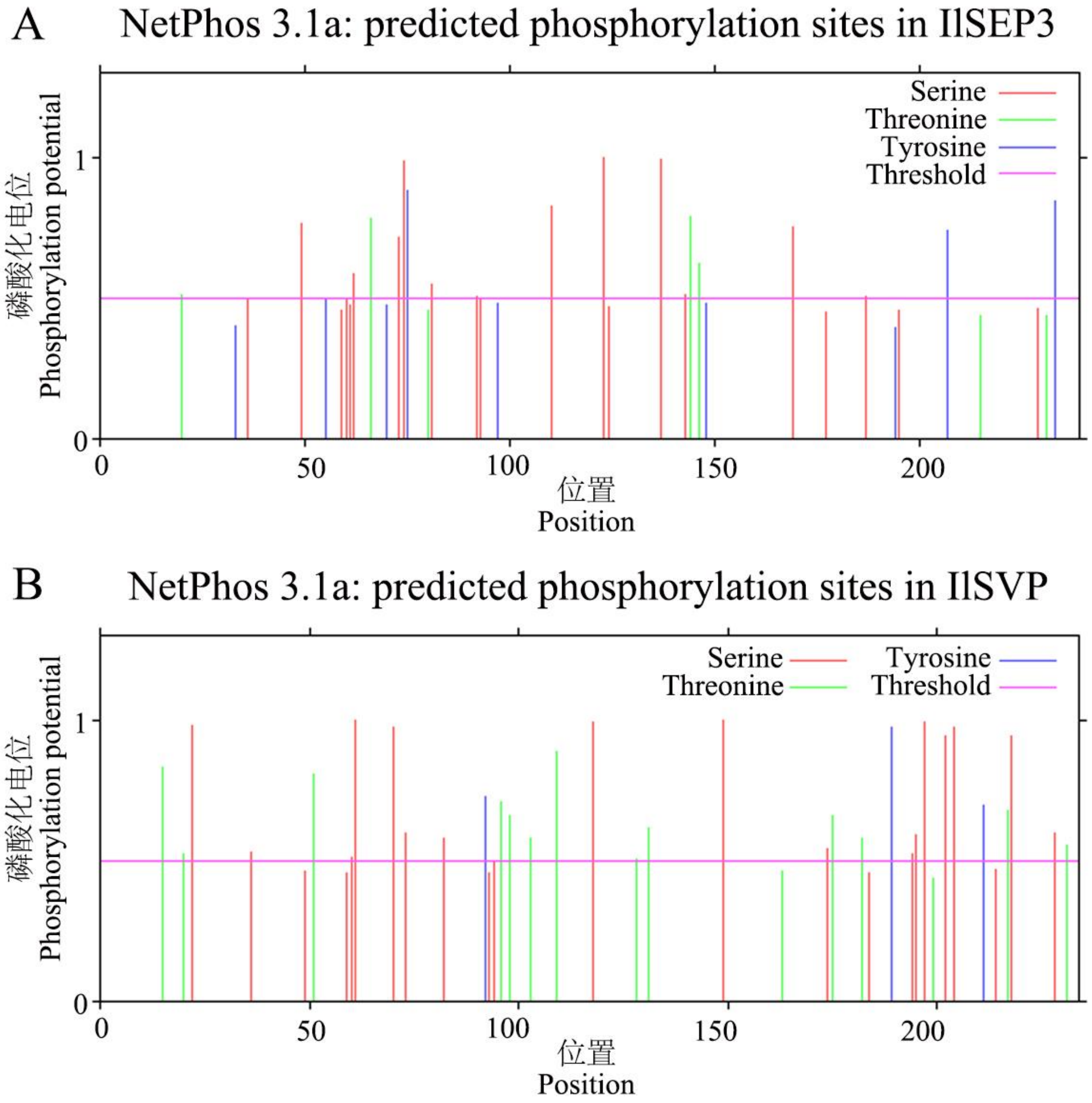

Supplemental Figure S8

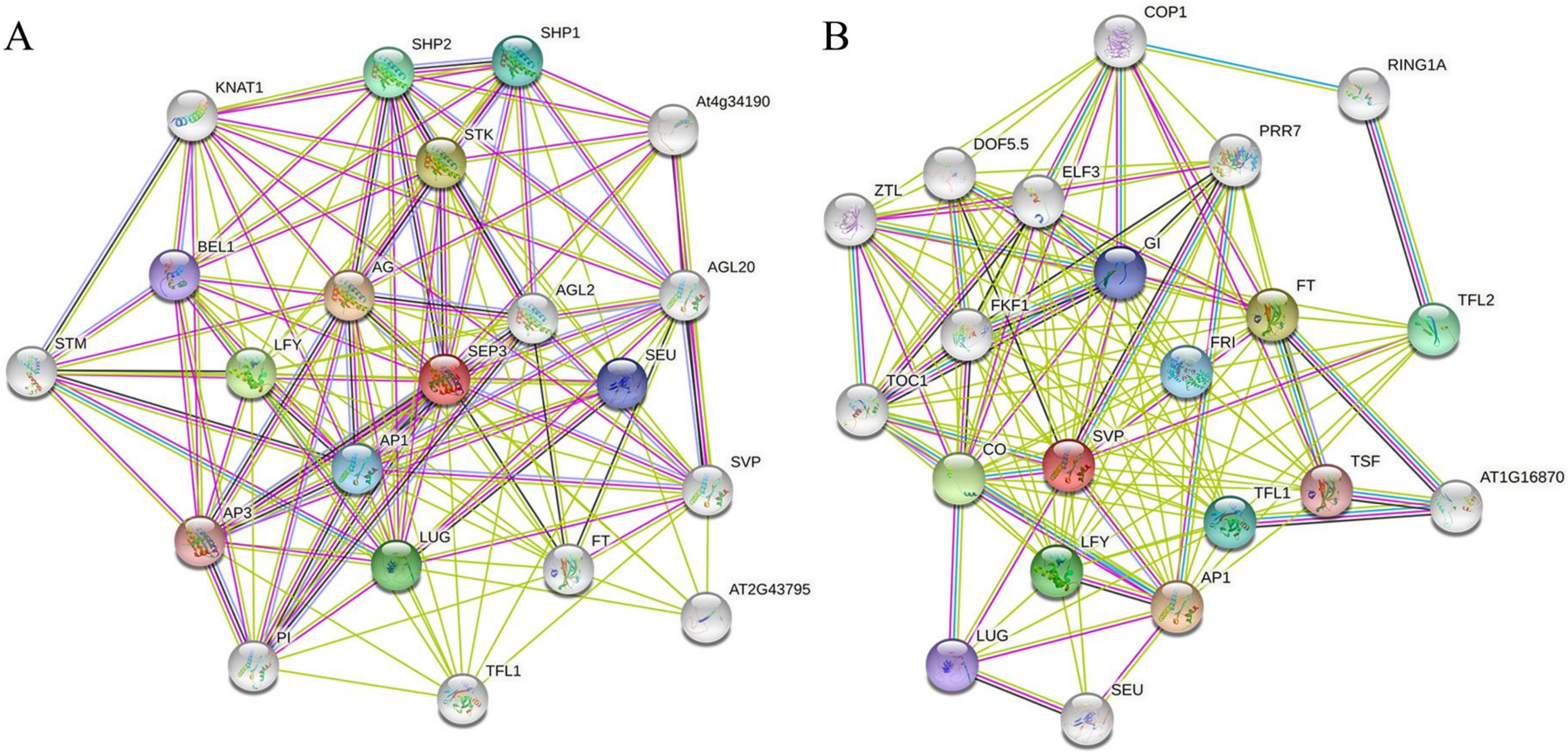

Supplemental Figure S9

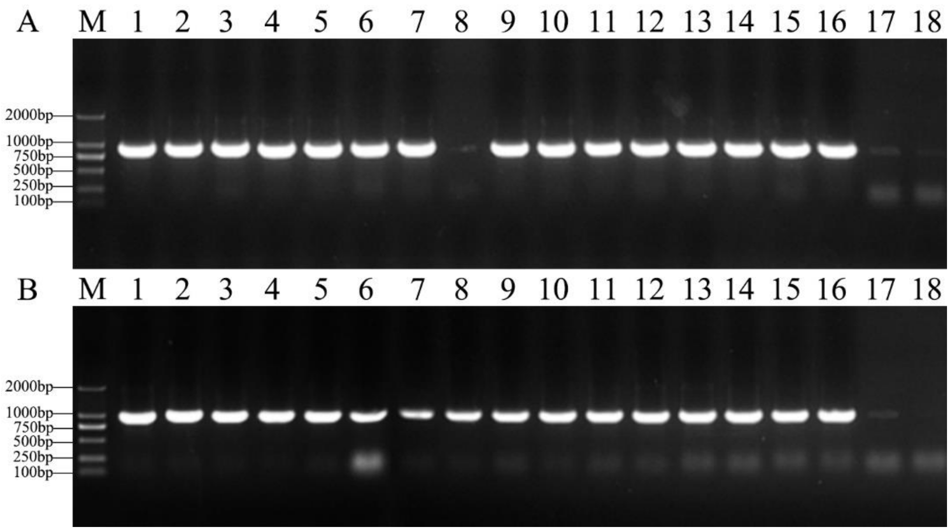

Supplement: Supplementary file 1 [file ijms-23-09950-s001.zip › ijms-1860119-supplementary figures.pdf]
