# Supplementary material for: Identification of MADS-Box Transcription Factors in Iris laevigata and Functional Assessment of IlSEP3 and IlSVP during Flowering
Source: Int J Mol Sci. 2022 Sep 1;23(17):9950. doi: 10.3390/ijms23179950 (PMC9456522; doi:10.3390/ijms23179950)
Supplement: Supplementary file 1 [file ijms-23-09950-s001.zip › ijms-1860119-supplementary table.pdf]

Table S1. Predicted physicochemical properties of *Il*SEP3 and *Il*SVP

	*Il*SEP3	*Il*SVP
ORF (bp)	720	705
Amino acid Residues	239	234
MW (kDa)	27.64	26.26
pI	8.99	6.24
Amino Acid composition	Ala (A) 5.9%、Arg (R) 8.4%、Asn (N) 4.6%、Asp (D) 4.2%、Cys (C) 1.7%、Gln (Q) 11.7%、Glu (E) 7.9%、Gly (G) 4.6%、His (H) 0.8%、Ile (I) 3.3%、Leu (L) 13.0%、Lys (K) 5.9%、Met (M) 2.1%、Phe (F) 2.1%、Pro (P) 3.3%、Ser (S) 8.8%、Thr (T) 2.9%、Trp (W) 0.8%、Tyr (Y) 3.8%、Val (V) 4.2%	Ala (A) 4.7%、Arg (R) 5.1%、Asn (N) 3.4%、Asp (D) 6.0%、Cys (C) 0.9%、Gln (Q) 6.8%、Glu (E) 9.4%、Gly (G) 6.8%、His (H) 0.9%、Ile (I) 4.3%、Leu (L) 11.5%、Lys (K) 9.8%、Met (M) 3.8%、Phe (F) 2.6%、Pro (P) 1.7%、Ser (S) 9.8%、Thr (T) 6.4%、Trp (W) 0.4%、Tyr (Y) 1.3%、Val (V) 4.3%
Instability index	52.53	52.76
Aliphatic index	81.63	78.76
GRAVY	-0.782	-0.676

ORF: open reading frame; MW: molecular weight; GRAVY: Grand average of hydropathicity.
